# Analysis of risk factors for radiation-induced oral mucositis for nasopharyngeal carcinoma and prognostic value of EGF and STREM-1

**DOI:** 10.5937/jomb0-49810

**Published:** 2025-01-24

**Authors:** Jiang Puyu, Xue Po, Wu Jiani

**Affiliations:** 1 Putuo District Central Hospital, Department of Otolaringology, Shanghai, China; 2 Hangzhou Traditional Chinese Medicine Hospital Affiliated to Zhejiang Chinese Medical University, Department of Otolaryngology, Hangzhou City, China

**Keywords:** nasopharyngeal carcinoma, radiationinduced oral mucositis, risk factors, epidermal growth factor, soluble myeloid cell expression trigger receptor-1, disease severity, prognosis, nazofaringealni karcinom, oralni mukozitis indukovan radijacijom, faktori rizika, epidermalni faktor rasta, rastvorljivi okidač ekspresije receptora mijeloidnih ćelija-1, ozbiljnost bolesti, prognoza

## Abstract

**Background:**

This study analyzed the risk factors of radiation-induced oral mucositis (RIOM) for nasopharyngeal carcinoma (NPC) and the correlation between epidermal growth factor (EGF), soluble myeloid cell expression trigger receptor-1 (sTREM-1), and disease severity.

**Methods:**

A total of 124 patients with NPC who received radiotherapy from March 2013 to November 2016 were enrolled and divided into the study group (n=68) and the control group (n=56) regarding the presence of RIOM. The risk factors of RIOM were evaluated by multivariate logistic regression. According to the severity of RIOM, patients who developed RIOM were divided into mild and severe groups, and the correlation between EGF, sTREM-1 and the severity of RIOM was analyzed by linear regression. According to the 5-year survival of patients after treatment, they were divided into a poor prognosis group (death, recurrence, and distant metastasis) and a better prognosis group and the predictive value of the prognosis of patients was analyzed by the ROC curve.

**Results:**

Age (>55 years), smoking, poor oral hygiene, and oral pH<7 were risk factors for RIOM, and the use of oral mucosal protective agents was a protective factor (P<0.05). In patients who developed RIOM, EGF levels decreased with increasing severity of RIOM, and sTREM-1 levels increased (P<0.05). The EGF level of patients was negatively correlated with the severity of RIOM. In addition, EGF levels in the poor prognosis group were lower than those in the better prognosis group, whereas sTREM-1 levels were higher than those in the better prognosis group (P<0.05). The AUC of the combined EGF and sTREM-1 levels test for predicting a patient's prognosis was greater than that of sTREM-1 alone (P<0.05).

**Conclusions:**

In short, age (>55 years), smoking, poor oral hygiene, and oral PH<7 are risk factors for RIOM for NPC. The use of oral mucosal protective agents is a protective factor. EGF and sTREM-1 levels are associated with RIOM severity and indicate predictive values for patient outcomes. This study provides new ideas for mitigating the occurrence of RIOM after radiotherapy to treat NPC.

## Abstract (sr)

**Uvod:**

Ova studija je analizirala faktore rizika za radijacionoindukovani oralni mukozitis (RIOM) kod nazofaringealnog karcinoma (NPC) i korelaciju između epidermalnog faktora rasta (EGF), rastvorljivog okidača ekspresije receptora mijeloidnih čelija-1 (sTREM-1) i ozbiljnosti bolesti.

**Metode:**

Uključeno je ukupno 124 pacijenta sa nazofaringealnim karcinomom (NPC) koji su primali radioterapiju od marta 2013. do novembra 2016. godine. Oni su podeljeni u studijsku grupu (n = 68) i kontrolnu grupu (n = 56) u zavisnosti od prisustva RIOM-a. Faktori rizika za RIOM su procenjeni pomoču multivarijantne logističke regresije. Prema ozbiljnosti RIOM-a, pacijenti koji su razvili RIOM podeljeni su u grupe sa blagim i teškim oblikom, a korelacija između EGF, sTREM-1 i ozbiljnosti RIOM-a je ana I izirana linearnom regresijom. Na osnovu petogodišnje stope preživljavanja pacijenata nakon lečenja, oni su podeljeni u grupu sa lošom prognozom (smrt, recidiv i udaljene metastaze) i grupu sa boljom prognozom, a prediktivna vrednost prognoze pacijenata analizirana je pomoču ROC krive.

**Rezultati:**

Faktori rizika za RIOM su bili godine (>55 godina), pušenje, loša oralna higijena i pH vrednost usne šupljine <7, dok je upotreba zaštitnih sredstava za oralnu sluzokožu bila zaštitni faktor (P<0,05). Kod pacijenata koji su razvili RIOM, nivoi EGF-a su se smanjivali sa porastom ozbiljnosti RIOM-a, dok su nivoi sTREM-1 rasli (P<0,05). Nivo EGF-a kod pacijenata bio je u negativnoj korelaciji sa ozbiljnošću RIOM-a. Pored toga, nivoi EGF-a u grupi sa lošom prognozom bili su niži nego u grupi sa boljom prognozom, dok su nivoi sTREM-1 bili viši nego u grupi sa boljom prognozom (P<0,05). AUC testa kombinovanih nivoa EGF-a i sTREM-1 za predviđanje prognoze pacijenta bio je veći od AUC testa samo sTREM-1 (P<0,05).

**Zaključak:**

Ukratko, starost (>55 godina), pušenje, loša oralna higijena i oralni pH<7 su faktori rizika za RIOM kod nazofaringealnog karcinoma (NPC). Upotreba zaštitnih sredstava za oralnu sluzokožu je zaštitni faktor. Nivoi EGF-a i sTREM-1 su povezani sa ozbiljnošću RIOM-a i ukazuju na prediktivne vrednosti za ishode pacijenata. Ova studija pruža nove ideje za smanjenje pojave RIOM-a nakon radioterapije za lečenje NPC-a.

## Introduction

Nasopharyngeal carcinoma (NPC) is one of the malignant tumours with the highest incidence in the department of otorhinolaryngology. Patients often have significant symptoms such as blood in the nose, tinnitus, and hearing loss, which can cause irreversible damage to their nasopharynx. Radiotherapy is commonly used in the treatment of NPC. Radiotherapy kills tumour cells but damages surrounding normal tissues, causing oral mucositis, mucous membrane ulcers to appear, erosion and so on, affecting the patient's eating normally [1] [2]. Radiation-induced oral mucositis (RIOM) is mainly caused by radiation directly, causing damage to the oral cell mucosa, affecting the DNA replication of mucosal cells and inhibiting their proliferation, destroying normal cell structure [3]. Poor oral hygiene can increase the risk of RIOM [4]. However, the risk factors for RIOM for NPC are still inconclusive. Epidermal growth factor (EGF) is a member of the growth factor family, which induces cell growth and migration and promotes the expression of differentiation genes. EGF can stimulate the growth and differentiation of oral mucosa, and the level of EGF in saliva is significantly reduced in the presence of inflammation in the oral cavity [5]. Furthermore, decreased EGF is directly associated with increased incidence and severity of mucositis [6]. In a study by Chen et al., heparin-bound epidermal growth factor (HB-EGF) was found to significantly increase epithelial thickness, neoepithelial cell division, and the quality and quantity of mucosal desmosomes in the oral cavity after radiation [7]. The soluble triggering receptor expressed on myeloid cell 1 (sTREM-1) is a new soluble inflammatory triggering receptor type. The signalling pathway mediated by it is involved in the occurrence and cascade of inflammatory responses. sTREM-1 has been extensively studied and appears to be a reliable biomarker of disease severity and outcome, especially in septic shock [8] [9]. In addition, the level of sTREM-1 is strongly correlated with the severity of radiological lung tissue injury [10]. However, its association with the progression of RIOM is unclear. Therefore, this study aimed to analyze the risk factors of radioactive oral mucositis for NPC, assess the correlation and prognostic value of EGF and sTREM-1 with disease severity, and provide a reference for the later development of NPC interventions and disease assessment.

### Data and methods

### Clinical data

A total of 124 patients with NPC who underwent radiotherapy from March 2013 to November 2016 were enrolled and divided into a study group (n = 68) and a control group (n = 56) regarding the presence of RIOM.

Inclusion criteria: patients meeting the diagnostic criteria for NPC in Practical Clinical Diagnosis and Treatment of NPC [11]; patients who were treated with radiation; patients without distant metastatic tumour lesions.

Exclusion criteria: patients with other malignant tumours; patients with distant metastasis of NPC; patients with oral ulcers, infection, and other oral problems before radiotherapy; patients with severe mental illness; patients with oral mucosal damage, swelling, and other phenomena or oral dysfunction before radiotherapy. 

## Material and methods

### Clinical data collection

The clinical data of the patients were collected using the clinical data questionnaire, which included age, body mass index, medical payment method, etc. Cigarette smoking was defined in cases when more than 1 cigarette was smoked per day for more than 6 months, and alcohol consumption was defined as more than or equal to 500 mL of beer and 200 mL of liquor or wine for more than 6 months. The normal body mass index (BMI) range was 18.5-23.9 kg/m^2^, with an abnormal range of <18.5 kg/m^2^ or >23.9 kg/m^2^.

### Radiotherapy

All patients were given radiotherapy. The radiotherapy target area was determined according to the size and extent of the tumour, and 7 common fields were irradiated. The target dose of primary tumour lesions was 2.12 Gy/F, and the total dose of the clinical tumour target area was 70 Gy/33F. Radiation was performed 5 times/week. The dose of the clinical prevention target area was 1.8Gy/F, and the total radiation dose was 50.4-59.6 Gy/28 33F. Radiation was performed 5 times a week.

### Grading of RIOM

According to the »Radiotherapy of Oncology« [12], the mucosal reaction was graded. Grade 0: absence of mucosal response and obvious symptoms; Grade 1: Oral mucosa congestion, redness, and swelling accompanied by mild pain; Grade 2: Oral mucosa congestion, redness, swelling, and punctate ulcers; Grade 3: Oral mucosa congestion, redness, and swelling, characterized by a flaky ulcer with albuginea and moderate pain impacting eating; Grade 4: The oral mucosa is extensive with ulcer or necrosis, making it challenging to open the mouth for eating. Mild mucositis is denoted by grade 1, while grades 2 to 4 signify severe mucositis.

### Detection of EGF and sTREM-1 levels

The researchers instructed the patients not to swallow for 1 minute and then coughed up the pooled saliva into a 50-mL tube to obtain a saliva sample. In addition, peripheral blood samples were obtained. The saliva and blood samples were centrifuged to separate the cellular debris and then frozen at -80°C until analyzed. The patient's saliva EGF level and serum sTREM-1 level were detected by enzyme-linked immunosorbent assay kits (Quantikine, R&D Systems).

### Prognosis

According to the 5-year survival of patients, they were divided into a poor prognosis group (death, recurrence, and distant metastasis) and a better prognosis group.

### Observation indicators

1. The general data and clinical data were com-1 pared, and the risk factors for RIOM were analyzed.

2. The levels of EGF and sTREM-1 of patients 2 were tested, and their correlation with the severity of RIOM and their predictive value on the prognosis of patients with NPC were analyzed.

### Statistical analysis

Data analysis was performed using SPSS22.0 software, and enumeration data (%) were compared by χ^2^ test. After checking the data normality, measurement data were expressed by mean ± standard deviation and evaluated by t-test. The risk factors of RIOM were assessed by multivariate logistic regression analysis, the prognostic value of EGF and sTREM-1 for RIOM was tested by ROC curve, and correlation analysis of the relationship between EGF and sTREM-1 levels and RIOM severity was by Spearman test. *P*<0.05 represented the significant difference.

## Results

### Univariate analysis of RIOM for NPC

The proportion of patients in the study group was higher than that in the control group in terms of age >55 years, smoking, TNM stage III-IV, poor oral hygiene, and oral PH<7. The proportion of patients receiving mucosal protective agents was lower than that in the control group (*P*<0.05, Table 1).

**Table 1:** Multivariate analysis of RIOM for NPC.

Factors		Study group<br>(n = 68)	Control group<br>(n = 56)	*χ^2^*	*P*
Gender	Male	39 (57.35)	29 (51.79)	0.384	0.535
	Female	29 (42.65)	27 (48.21)		
Age (Years)	>55	35 (51.47)	17 (30.36)	5.622	0.018
	55	33 (48.53)	39 (69.64)		
Body mass index	Normal	46 (67.65)	37 (66.07)	0.034	0.853
	Abnormal	22 (32.35)	19 (33.93)		
Smoking		29 (42.65)	12 (21.43)	6.247	0.012
Drinking		16 (23.53)	11 (19.64)	0.272	0.602
Payment	At own expense	12 (17.65)	8 (14.29)	0.256	0.613
	Medical insurance	56 (82.35)	48 (85.71)		
Education	Junior high school and below	45 (66.18)	41 (73.21)	0.716	0.398
	High school and above	23 (33.82)	15 (26.79)		
Marriage	Married	51 (75.00)	43 (76.79)	0.053	0.817
	Unmarried or divorced, widowed	17 (25.00)	13 (23.21)		
TNM	I~II	37 (54.41)	42 (75.00)	5.63	0.018
	III~IV	31 (45.59)	14 (25.00)		
Oral hygiene	Excellent	32 (47.06)	39 (69.64)	6.4	0.011
	Poor	36 (52.94)	17 (30.36)		
Oral mucosal protectant		36 (52.94)	41 (73.21)	5.363	0.021
Antibiotics before radiotherapy		41 (60.29)	42 (75.00)	3.001	0.083
Combined chemotherapy		61 (89.71)	12 (21.43)	59.126	0
Combined diabetes		10 (14.71)	7 (12.50)	0.126	0.722
Oral PH	<7	24 (35.29)	10 (17.86)	4.692	0.03
	≥7	44 (64.71)	46 (82.14)		

### Multivariate analysis of RIOM for NPC

Multifactor logistic regression analysis found that age (>55 years) (*OR*=2.321, *P*=0.003), smoking (*OR*=3.161, *P*=0.004), poor oral hygiene (*OR*=1.861, *P*=0.045), and oral pH<7 (*O*=2.186, *P*=0.002) were risk factors for RIOM for NPC, and oral mucosal protective agents indicated a protective factor (*OR*=0.550, *P*=0.006, Table 2).

**Table 2:** Multivariate analysis of RIOM for NPC. Assignment: occurrence of radiation oral mucositis (1 for yes, 0 for no); age (1 for >55 years, 0 for <55 years old); smoking (1 for yes, 0 for no); TNM stage (stage III–IV); Oral hygiene (1 for poor, 0 for excellent); oral pH (1 for 7, 0 for <7); use of oral mucosal protective agents (1 for yes, 0 for no).

Factors	β	SE	wald χ^2^	OR	95%CI	P
Age	0.842	0.286	8.667	2.321	1.325~4.066	0.003
Smoking	1.151	0.397	8.406	3.161	1.452~6.883	0.004
TNM	0.416	0.243	2.931	1.516	0.942~2.441	0.088
Oral hygiene	0.621	0.309	4.039	1.861	1.015~3.410	0.045
Oral PH	0.782	0.246	10.105	2.186	1.350~3.540	0.002
Oral mucosal protective agent	-0.597	0.215	7.71	0.55	0.361~0.839	0.006

### Comparison of the levels of EGF and sTREM-1

**Figure 1:**
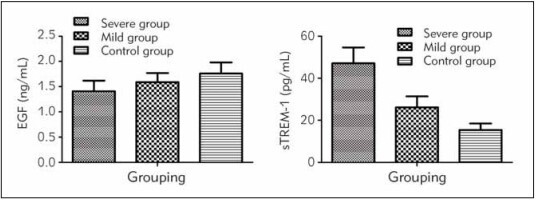
Comparison of EGF and sTREM-1 levels in groups.

The level of EGF decreased while that of sTREM-1 increased with the severity of RIOM (*P*<0.05, Figure 1).

### Correlation analysis between the levels of EGF and sTREM-1 and the severity of RIOM

**Figure 2:**
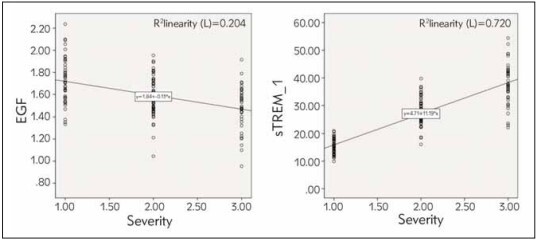
Correlation analysis between the levels of EGF and sTREM-1 and the severity of RIOM.

EGF levels showed a negative correlation with the severity of RIOM (y=1.84-0.13*x), whereas sTREM-1 levels exhibited a positive correlation (y=4.71+11.19*x) (*P*<0.05, Figure 2).

### Levels of EGF and sTREM-1 in patients with different prognoses 

**Figure 3:**
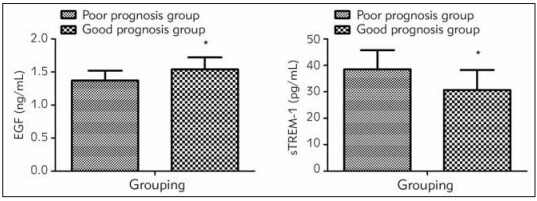
Levels of EGF and sTREM-1 in patients with different prognoses. * P<0.05 vs. the poor prognosis group.

Lower EGF level and higher sTREM-1 level were measured in the poor prognosis group rather than the better prognosis group (*P*<0.05, Figure 3). 

### Prognostic value of EGF and sTREM-1 levels 

**Figure 4:**
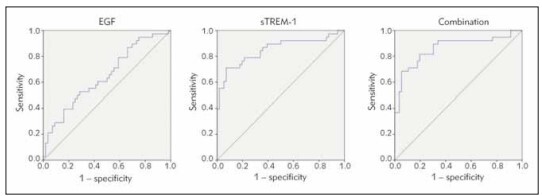
ROC curve analysis of EGF and sTREM-1 levels in predicting the prognosis of patients.

AUC of EGF and sTREM-1 levels (AUC=0.862, 95%CI:0.779~0.946) in predicting the prognosis of patients was greater than that of sTREM-1 alone (AUC=0.648, 95%CI:0.534~0.761) (*P*<0.05, Table 3, Figure 4).

**Table 3:** Prognostic value of EGF and sTREM-1 levels.

Indicators	Cutoff value	AUC	SE	95% CI
EGF	1.46 ng/mL	0.85	0.045	0.763~0.938
sTREM-1	34.92 pg/mL	0.648*	0.058	0.534~00.761
Combined use		0.862	0.043	0.779~00.946

## Discussion

RIOM is a common dose-limiting toxic reaction during radiotherapy for head and neck tumours, which refers to the oral mucosal damage caused by the stimulation of ionizing radiation, mainly manifested as oral mucosal congestion, ulceration and even decay, accompanied by pain and difficulty in eating, which has a serious impact on the quality of life of the patients and their tolerance of radiotherapy [13] [14]. By analyzing the risk factors affecting RIOM, reasonable interventions can be given [15]. At present, it is believed that reducing immune function increases the risk of RIOM. During the process of radiotherapy, the normal immune function of the patient gradually declines, and the secretion of neutrophils in the body decreases, which leads to the proliferation of oral bacteria and, eventually, oral mucositis [16] [17]. Oral hygiene is also related to the occurrence of RIOM [18]. There are many clinical reports on the risk factors for RIOM, but the demographics and basic conditions of patients in different hospitals in different regions are also different, so there may be differences in the risk factors affecting patients with RIOM. Therefore, according to the basic situation of patients in Shanghai Ninth People's Hospital, Shanghai JiaoTong University School of Medicine, this study conducted a correlation analysis on the influencing factors of RIOM in patients. The results showed that age (>55 years), smoking, poor oral hygiene, and oral PH<7 were the main causes of RIOM for NPC, and the administration with oral mucosal protective agents was of protection, which is mainly due to oral infections in patients with poor oral hygiene the probability of increasing. Toxic substances in tobacco can stimulate oral mucosa, resulting in a decrease in the proliferation of oral mucosa epithelial cells, and the high temperature during smoking can burn the contact part of the oral mucosa. In addition, smoking can affect local blood circulation and humoral immune processes, indirectly increasing the risk of RIOM [19]. Chemotherapy is also a risk factor for oral mucositis in patients with NPC [20], which can cause bone marrow suppression, leukopenia, and decreased immunity. However, our study results highlighted that combined chemotherapy did not increase the risk of the disease in patients, which may be related to the uneven proportion of chemotherapy patients and non-chemotherapy patients and the small sample size, so the sample size needs to be increased for further analysis. 

sTREM-1 is mainly expressed in neutrophils, monocytes and macrophages and can promote the release of inflammatory mediators [21]. Activation of sTREM-1 could induce the production of pro-inflammatory factors [22]. sTREM-1 can bind to transmembrane proteins through the intracellular short tail region and its corresponding receptors, activating downstream signalling pathways and enabling many inflammatory mediators to be synthesized and released [23] [24]. EGF is mainly synthesized and secreted by acinar cells and duct epithelial cells of salivary glands in the body. It is widely present in human and animal tissues and body fluids. Salivary gland damage can lead to a decrease in the concentration of EGF in saliva [25].

EGF in saliva plays an important role in maintaining the integrity of the mucosal epithelial barrier and promoting the healing of mucosal injury. Our findings evidenced that The level of EGF in patients was negatively correlated with the severity of radiation oral mucositis, and the level of sTREM-1 was positively correlated with it. This is mainly related to the fact that the decrease of EGF can affect the oral mucosal function, and the increase in the level of sTREM-1 can aggravate the local inflammatory response [26] [27]. A report has implied that sTREM-1 levels can be used to evaluate the prognosis of patients with infectious diseases [28]. This study found that the lower EGF level and higher sTREM-1 were seen in patients with poor prognosis, suggesting that EGF and sTREM-1 may be useful in evaluating the prognosis of NPC patients with RIOM. Also, our research clarified that the AUC of EGF and sTREM-1 levels in predicting the prognosis of patients was greater than that of sTREM-1 alone, indicating that the combined detection of EGF and sTREM-1 levels have predictive value for the prognosis of patients with NPC.

In conclusion, age >55 years, smoking, poor oral hygiene, and oral pH<7 are risk factors for RIOM for NPC. The use of oral mucosal protective agents is a protective factor. EGF and sTREM-1 levels correlate with RIOM severity and have predictive values for patients' prognosis. Adopting individualized preventive programs to address the risk factors of different patients is vital to reduce the incidence of RIOM.

## Dodatak

### Acknowledgements

Not applicable.

### Funding

Not applicable.

### Availability of data and materials

The datasets used and/or analyzed during the present study are available from the corresponding author upon reasonable request.

### Ethics approval

The present study was approved by the Ethics Committee of Shanghai Ninth People’s Hospital, Shanghai JiaoTong University School of Medicine and written informed consent was provided by all patients before the start of the study. All procedures were performed following the ethical standards of the Institutional Review Board and The Declaration of Helsinki, as well as its later amendments or comparable ethical standards.

### Authors’ contributions

Puyu Jiang and Po Xue designed the research study. Puyu Jiang performed the research. Po Xue provided help and advice on the experiments. Jiani Wu analyzed the data. Puyu Jiang and Po Xue wrote the manuscript. All authors contributed to editorial changes in the manuscript. All authors read and approved the final manuscript.

### Conflict of interest statement

All the authors declare that they have no conflict of interest in this work.
